# Improving Manufacturing Applications of Machine Learning by Understanding Defect Classification and the Critical Error Threshold

**DOI:** 10.1007/s40962-021-00637-0

**Published:** 2021-06-24

**Authors:** David Blondheim

**Affiliations:** 1 Colorado State University, Fort Collins, CO USA; 2 Mercury Marine—Mercury Castings, A Division of Brunswick, Inc., Fond du Lac, WI USA

**Keywords:** supervised machine learning, machine learning, classification issues, misclassification, bias error, inherent error, critical error threshold, porosity, artificial intelligence (AI), casting defects, defect classification, high-pressure die casting (HPDC), manufacturing, unsupervised machine learning

## Abstract

Machine learning (ML) is unlocking patterns and insight into data to provide financial value and knowledge for organizations. Use of machine learning in manufacturing environments is increasing, yet sometimes these applications fail to produce meaningful results. A critical review of how defects are classified is needed to appropriately apply machine learning in a production foundry and other manufacturing processes. Four elements associated with defect classification are proposed: *Binary Acceptance Specifications, Stochastic Formation of Defects, Secondary Process Variation,* and *Visual Defect Inspection.* These four elements create data space overlap, which influences the bias associated with training supervised machine learning algorithms. If this influence is significant enough, the predicted error of the model exceeds a critical error threshold (CET). There is no financial motivation to implement the ML model in the manufacturing environment if its error is greater than the CET. The goal is to bring awareness to these four elements, define the critical error threshold, and offer guidance and future study recommendations on data collection and machine learning that will increase the success of ML within manufacturing.

## Introduction

Machine learning (ML), a specific subset of technology in the Artificial Intelligence (AI) field, has seen an explosion in commercial use in the past 30 years. From utilizing computer vision to recognize hand-written numbers for the post office in the 1990s^1^ to current applications to trace and treat the COVID-19 pandemic,^2^ the ability of machine learning to unlock patterns and provide insight into data has provided great benefits to society.

Machine learning has also found increased use in manufacturing and operational systems. Industry 4.0 and Smart Manufacturing have driven digitalization of manufacturing operations. Microsoft and Amazon have made large investments in this technology as they partner with manufacturers to provide data collection and analytics tools.^3^^,^^4^ Estimates show a 10.1% compounded annual growth rate of investment into smart manufacturing technologies with a 2024 global spend rate predicted at $400 billion USD.^5^

This growth of ML within manufacturing comes with trepidation by industry leaders. A 2021 survey by KMPG^6^ shows 55% of industrial manufacturing business leaders believed AI adoption within manufacturing is moving faster than it should. Within this same survey, the industrial manufacturing segment had the largest number of respondents who believed that AI initiatives delivered somewhat or significantly less value within the organization, with more than 21% falling in these two categories. There is a considerable challenge to implement ML in industrial settings. Experience has shown many software providers oversell the benefits of ML/AI and downplay the effort needed to implement ML to an organization’s executives. The effort to successfully implement ML should not be underestimated. Many ML projects in manufacturing fail to provide value. Unfortunately, the KMPG survey highlights the gap that exists in leadership ranks of organizations. Seventy-eight percent of executives found value within AI initiatives, while only 50% of managers reported finding the same value in these projects. This work will show how misclassifications of product within production environments can assist with these ML failures.

Beyond the leadership survey, research has also highlighted the challenges with applying ML in manufacturing. Wuest et al.^7^ describe challenges that manufactures face with ML, including the acquisition and pre-processing of data; high dimensionality of manufacturing data; highly unbalanced data sets; selection of ML algorithms; and interpretation of the results. Baier et al.^8^ interviewed multiple industries, including manufacturing, to identify the most significant challenges in implementing ML. They identified challenges existing in three key areas:

1. Pre-deployment stage: companies must gather the right quality data in sufficient amounts.
2. Deployment stage: challenges are associated with gathering large amounts of data and ensuring hardware and software systems can handle the volume of data.
3. Non-technical items: acceptance of ML models for people who have no background in data science and people questioning the practical value ML can provide in manufacturing.

The pre-deployment stage is critical to ensure the data used for ML training is correct. Misclassification of training data can lead to errors, specifically within supervised ML algorithms. Supervised and unsupervised ML are the most used algorithm types in ML applications. In supervised ML, algorithms are trained from labeled data sets. Multiple input values are provided to a supervised ML algorithm to find a pattern to help accurately predict a future result. The algorithm is trained on both the inputs and results. It is then tested against a data set that the algorithm was not trained on to see how well the model can predict results.^9^,^10^ As will be discussed, misclassification of products occurs in production manufacturing environments. This noise introduced into the supervised ML algorithm leads to poor modeling, prediction failures, and ultimately ML not being implemented. In unsupervised ML, no results are known in the data sets. Only the input parameters are provided for the algorithm to detect patterns. Unsupervised ML focuses on clustering analysis and anomaly detection.^9^,^10^ The focus of this work is on supervised ML as it is commonly used in manufacturing applications of ML. Supervised ML would be used in classification predictions, such as predicting part quality. However, due to some of the shortcomings associated with misclassification of data used in manufacturing, applications of unsupervised ML will be discussed in the recommendations.

Challenges with applying ML in the metal casting industry have also been studied. Sun et al.^11^ discusses unbalanced data sets, sporadically labeled data, and how metal casting data is not seen as the “big data” associated with ML given that some casting processes can take years to generate tens-of-thousands of rows of data. Traceability and classification of castings are challenges in a production environment.^12^ Classification of casting quality is of high interest when attempting to optimize the process within the foundry. The technical process to serialize parts is not as difficult as the actual process of collecting and tracing these parts through multiple manufacturing operations, facilities, and companies within the supply chain.^13^ Often a human is at the end of this process inspecting the casting and classifying the results against print specifications. Studies have shown that humans are inconsistent visual inspectors, missing 20% to 50% of defects in various manufacturing processes.^14^–^19^

**Figure 1:**
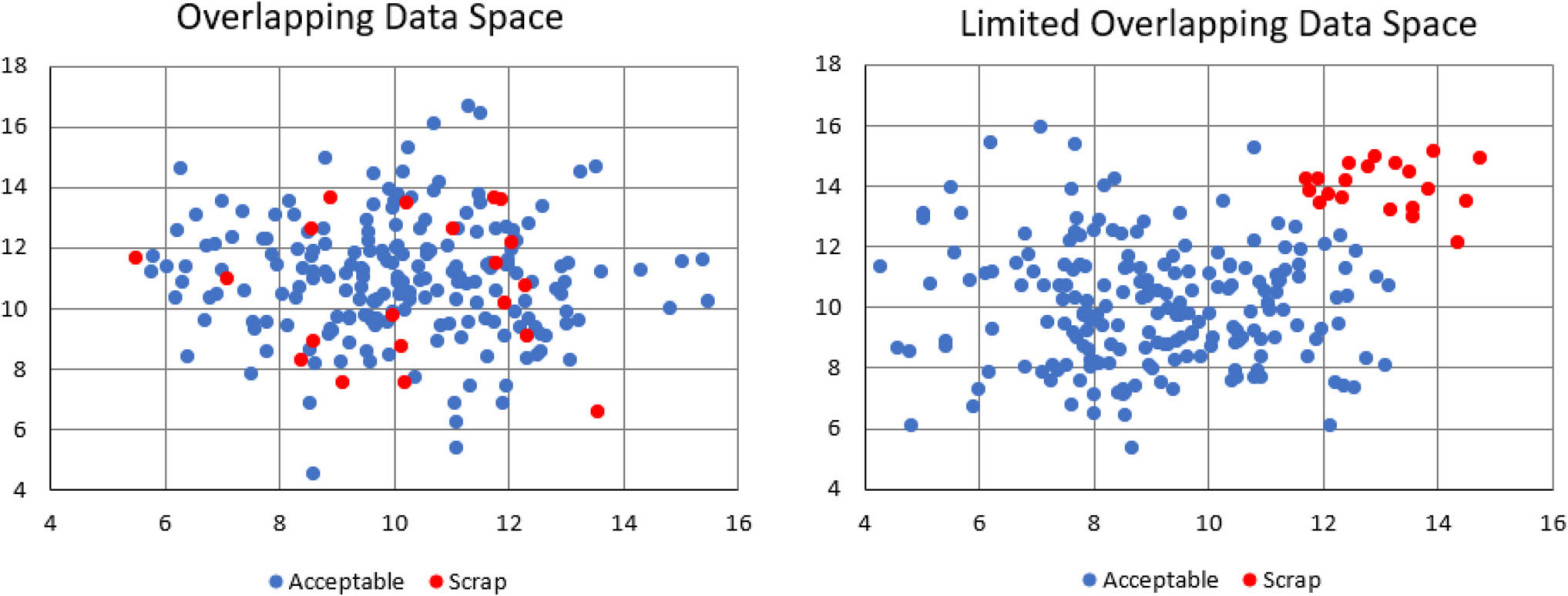
Example of overlapping data space.

Misclassification of results causes data space overlap in machine learning. This overlap can fundamentally diminish the results of supervised ML. Overlap occurs when multiple classifications of training data results occur in the same dimensional data space.^20^ A simple 2-dimensional example of data overlap is found in Figure 1. The left side of the figure shows why it is difficult for ML applications to find a pattern in noisy, overlapped data. ML can build models that accurately predict the situation with the limited overlap as seen on the right side of Figure 1. Misclassification of results, either done by human error or by process variation, is a significant contributor to overlap within manufacturing data sets.

This work will show how defect classification systems used in product manufacturing influence ML models. Misclassifications of defects can make applications of supervised ML exceed a critical error threshold (CET) rendering the model financially useless. Proposed are four elements of defect classifications in production environments that cause these issues for ML:

1. Binary acceptance specifications
2. Stochastic formation of defects
3. Secondary process variation
4. Visual defect inspection

These four elements will be reviewed from the high-pressure die casting (HPDC) perspective. Due to the modularity of these elements and the generality of the CET, these concepts have applications in other manufacturing processes such as sand casting, permanent mold casting, machining, painting, and assembly. Finally, the issues with the misclassification will be discussed in terms of data space overlap, which creates a bias shift while training supervised ML.

The goal is to help organizations understand and avoid the pitfalls commonly misclassified manufacturing data can have on ML. With this knowledge, organizations should drive to improve data classification process especially when there is an intention to implement ML. At a minimum, this work can provide insights into why some applications of ML may have failed in the past due to poor, but commonly used, practices of data collection in manufacturing.

## Background in High-Pressure Die Casting

High-pressure die casting (HPDC) is composed of multiple systems that control hydraulic, mechanical, and thermal processes to produce near net-shape castings with short cycle times in complex metal molds.^21^ HPDC typically focuses on large production volumes due to the capital investment in equipment, tooling costs, and short production cycle times.

The design of the casting and tooling, combined with the setting and control of the manufacturing system, dictates casting quality and equipment performance. The North American Die Casting Association (NADCA) estimates annual sales of $8 billion USD for aluminum die casting in 2019. This represents more than 80% of the American Foundry Society’s (AFS) forecast in all aluminum castings of $9.67 billion USD.^22^ Based on a 2014 NADCA study, current methods for control and optimization in HPDC produce a median scrap rate of 8% of parts produced and equipment utilization of 68% within the industry.^23^ Improving uptime and reducing scrap costs can create meaningful value.

The process associated with HPDC makes castings prone to defects. As aluminum turns from liquid to solid during the casting process, its volume shrinks by approximately 6%.^24^,^25^ If unfed, the casting will have void space called porosity. Porosity is typically found in the thickest portions in the casting. HPDC’s goal is to reduce this unfed area by applying high amounts of pressure on the liquid metal to continue feeding the casting during solidification.^21^,^26^ Casting geometry design, gating and die design, and equipment size all factor into how well this void space can be fed.^25^,^27^ In production, all castings will have some level of void space. It is the goal of the design and manufacturing engineers to place this in non-critical areas of the casting. Unfortunately, as seen in the 8% median scrap rate, porosity-related defects continue to challenge the quality and reliability of the HPDC process.

Porosity is just one of multiple potential defects in HPDC. Defects can be described both in the location as to where they form and the metallurgical cause. Multiple publications are available that describe different classification systems for HPDC defects in these terms.^28^–^30^ These defect classification systems are important to provide consistent levels of defect feedback to the foundry. It is difficult to successfully complete quality control and improvement projects without a common defect language.^30^ In addition to porosity, the other most common defect types in HPDC include laminations, inclusions, leaking, cracks, blisters, soldering, erosion, and deformation. Although all these defects are important, the focus of this work is on the porosity defects. Porosity is a leading cause of scrap within the die cast process.^28^ Approximately, 30% of the foundries within the industry have identified the need to address porosity as top concern.^31^ The HPDC community will see great value by applying ML to improve porosity and other quality defects.

**Figure 2:**
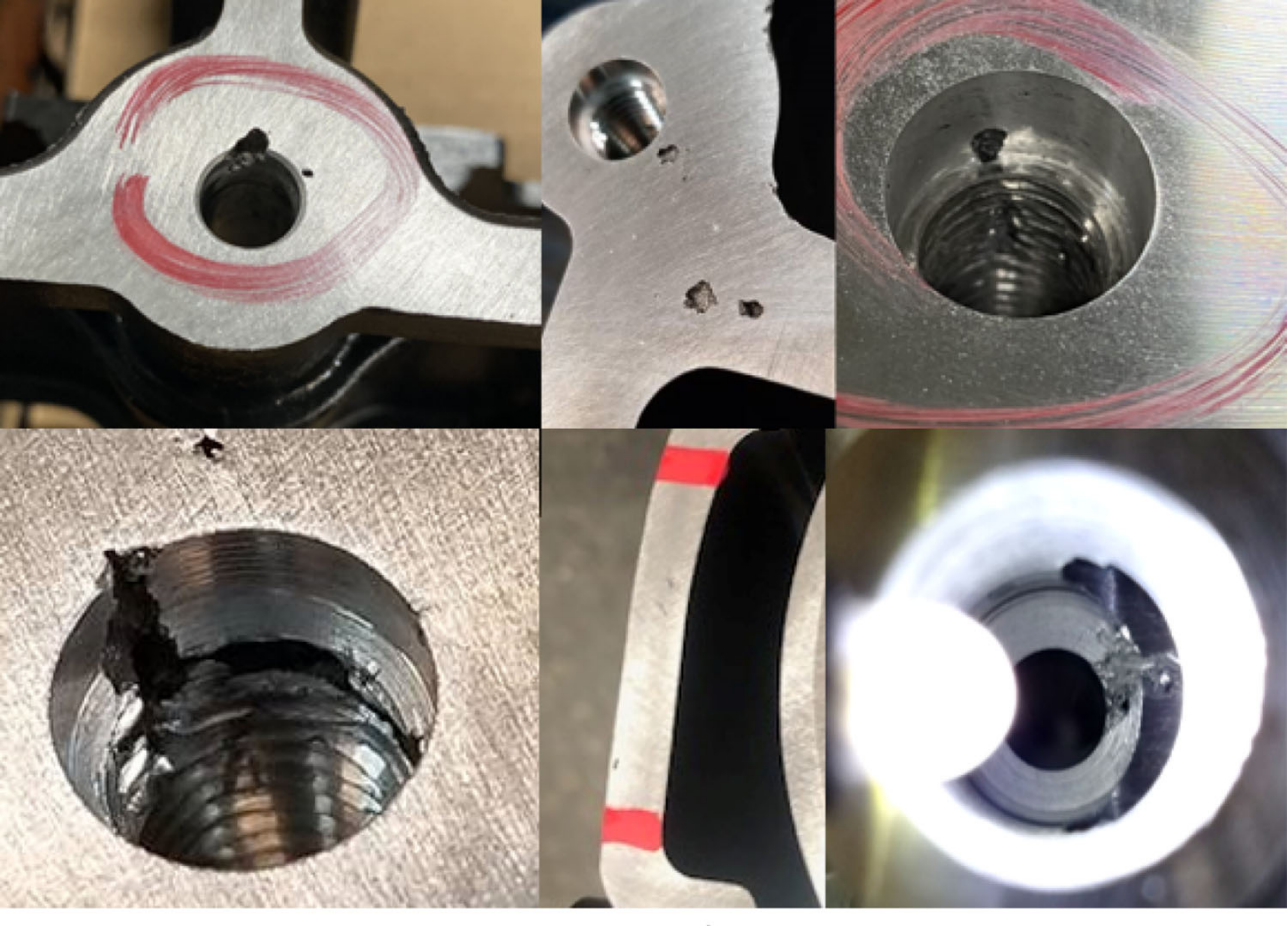
Examples of HPDC porosity.

Porosity that impacts the quality in production castings is created from entrapped gas, called gas porosity, or volumetric shrink, called shrink porosity. These two types of porosity can combine to form gas-assisted shrink. These defects’ causes and physical descriptions are well published.^24^–^28^ Production examples of porosity defects can be seen in Figure 2.

Porosity defects are commonly internal to the casting. As a result, the defect is usually uncovered after additional processing. This means manufacturing costs such as trimming, shot blasting, painting, machining, testing, and inspection are added to the casting cost prior to the defect being found. In addition to the costs, there is a time delay associated between casting production and when the feedback is received. In the ideal situation, this feedback time is extremely short due to single piece flow. In real-world manufacturing facilities, it is common for there to be several weeks, if not months, between casting production and defect feedback. Reasons for these delays include batch processing typical in manufacturing; complex, international supply chains; transportation time and safety stock levels; multiple vendors performing different manufacturing operations; and insufficient inspection and analysis approaches for product. To minimize this risk, foundries utilize casting simulations; experiments to help determine process settings and limits; and radiographic, or X-ray, audits to identify changes to the HPDC process which may result in increased porosity.

Casting simulations have proven to be successful at identifying high-risk areas of porosity within HPDC.^32^ By simulating the filling and solidification of the given casting geometry and die design, software can predict areas of entrapped gas and unfed shrink regions. These predicted areas represent a probability of where porosity is most likely to form in the casting. Ideally, these areas are eliminated by geometry changes in the casting, changes to the die’s gating or venting system, thermal management of the tool, or process changes on the injection of the metal.^33^–^36^ This cannot always be accomplished due to functional requirements of the final part assembly and process limitations. Since die cast tooling is so complex, once a die is created it is expensive, difficult, and risky to make changes to tooling while trying to support production volumes. Additionally, simulations are successful at identifying concern areas, but they are not 100% accurate due to the assumptions and generalizations made when setting the initial conditions of the simulation. As a result, the HPDC industry often works in less than ideal situations when trying to produce castings free of porosity.

**Figure 3:**
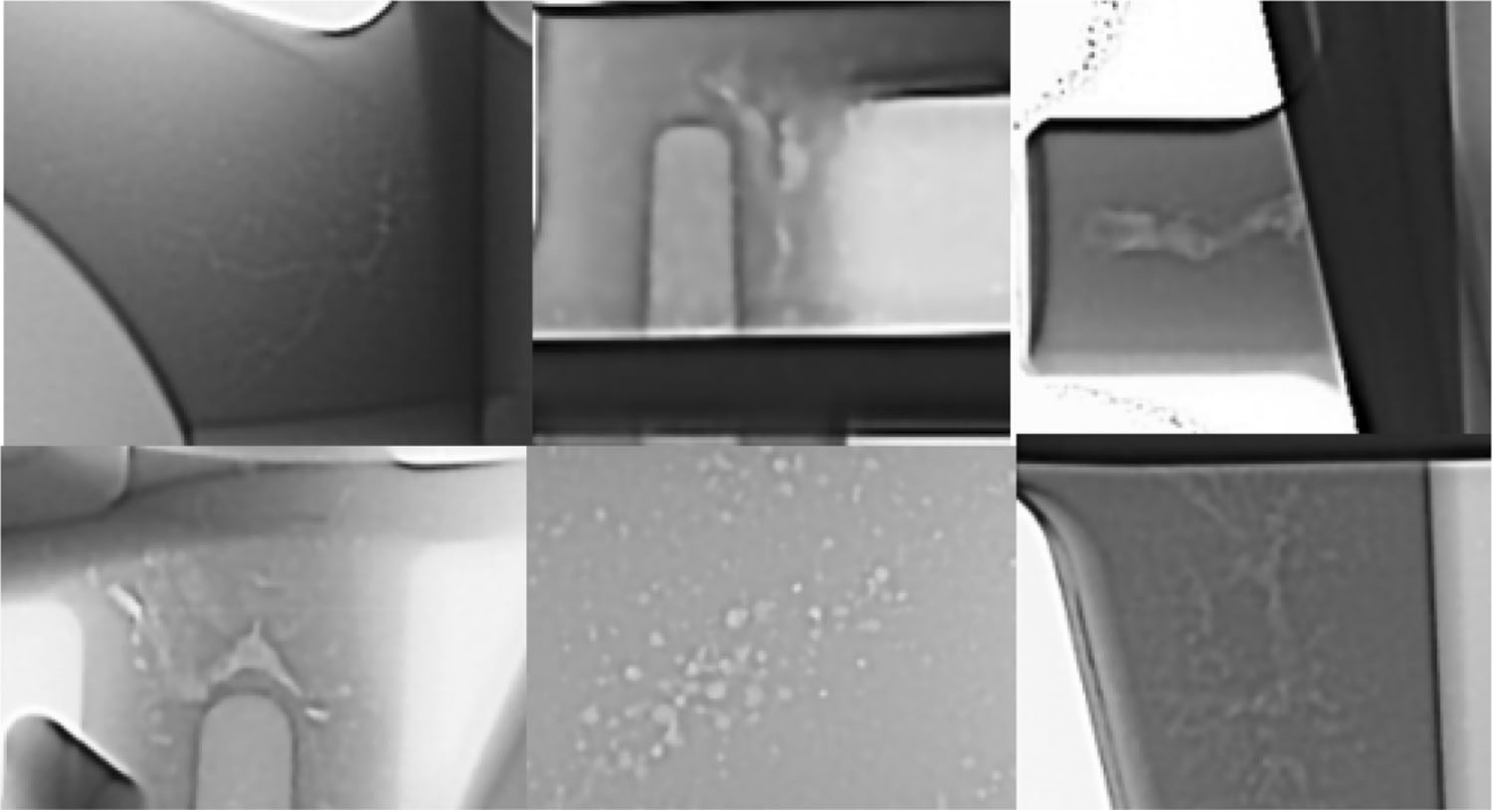
Sample X-Ray images of porosity.

Foundries will often X-ray inspect production castings to minimize the risk of creating castings with porosity. In the current industrial environment, usually only structural automotive^37^ or aerospace^38^ castings utilize 100% X-ray inspection. These types of castings can justify the added costs of equipment and processing time to inspect every produced casting in critical locations. In most other casting applications, the production cycle time, cost, or functional need creates an economic situation where castings will only be sampled for X-ray inspection. This audit will help identify changes in typical porosity levels, which may indicate special cause variation in the manufacturing process. Examples of porosity in X-ray images are seen in Figure 3.

Even by reducing the possibility of porosity with simulations and control of the process with X-ray, porosity defects still pass through the supply chain and are identified after additional value is added. Without 100% X-ray, porosity scrap is found after machining when the void is exposed with milling or drilling operations. After the machining operation is complete, human operators visually inspect the machined castings to determine if they pass a porosity specification. The decision made is binary. Castings will either be classified as *acceptable* or *scrap*. When a *scrap* part fails to meet porosity requirements, a repair method such as welding or epoxy may be allowed in certain applications. For this work, a focus will remain on the primary classifications of *acceptable* or *scrap* since the goal is to create ML predictions that would help eliminate poor quality regardless of its ability to be repaired. This binary classification of castings contributes to the issues with applying machine learning and will be discussed in detail in the next section.

## Four Elements of Defect Classification

In HPDC, obtaining accurate classifications is a challenge that needs to be fully understood. Misclassified training data has consequential impact on the training bias of supervised ML. There are four key elements of classification: *Binary Acceptance Specifications, Stochastic Formation of Defects, Secondary Process Variation,* and *Visual Defect Inspection.*

### Binary Acceptance Specifications

In Juran’s Quality Handbook, a defect is described as “anything that does not meet or exceed the requirements of the customer, the business, or the process” and states the “importance to have a realistic threshold for what is called a defect”.^14^ In manufacturing environments, this threshold is given as a quality specification for the product. The specification is typically set during the design and testing phases to ensure the product achieves the functionality intended.

A formal specification is included in manufacturing prints or as a stand-alone document to ensure conforming product is provided to the customer. This specification becomes an aid during the inspection and will include a threshold of acceptable level of defects. Parts that exceed this threshold are classified as *scrap*, while parts below are classified as *acceptable*. A binary classification is made.

**Figure 4:**
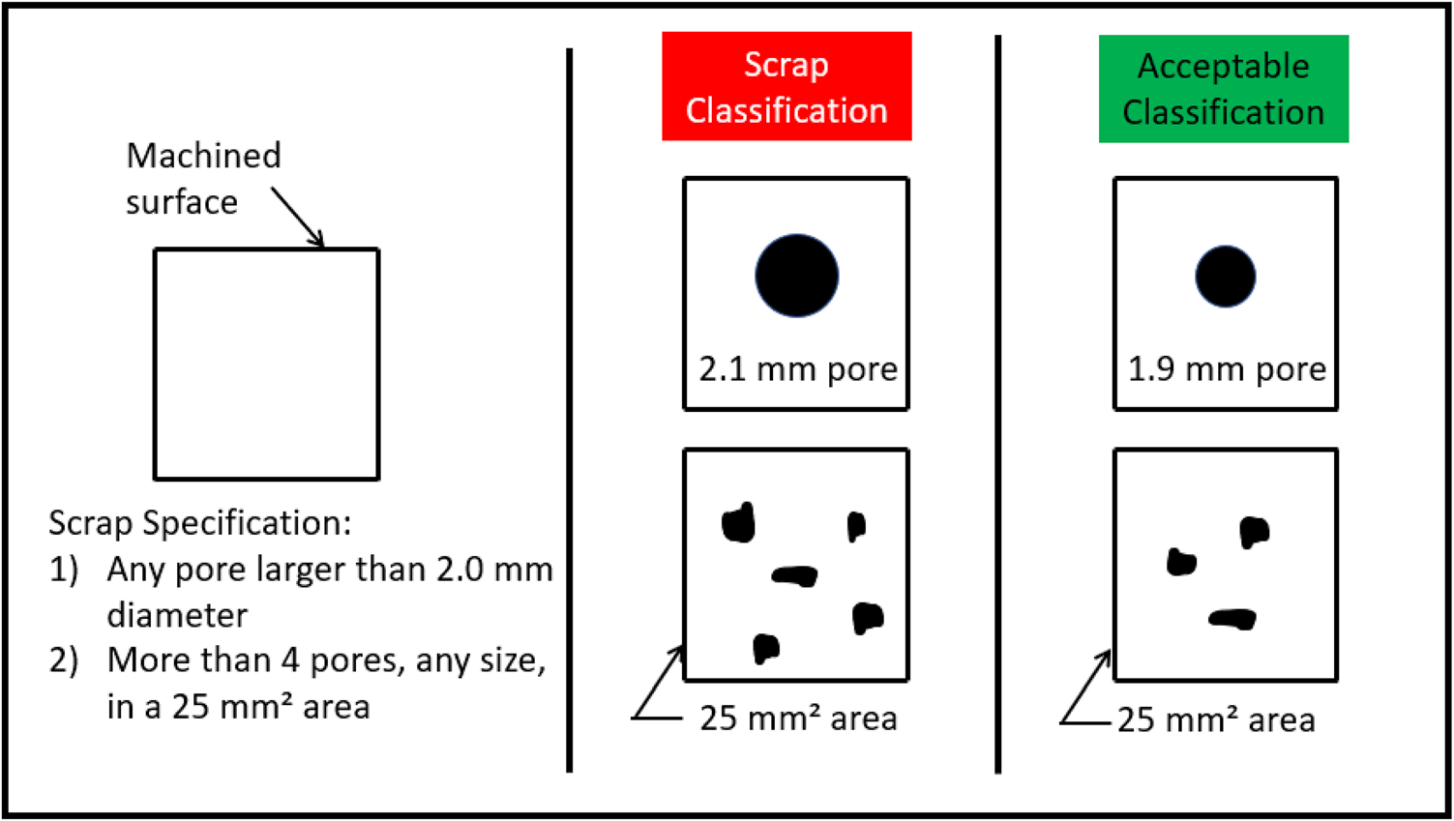
Classification issue due to binary acceptance specifications example.

A common approach used for porosity in a production environment is defining a maximum permissible porosity size and number of pores per region.^27^ Figure 4 shows an example of a theoretical specification for a casting based on a max pore size of 2.0 mm and a max allowable number of pores to be 4 in a 25 mm^2^ area.

In most applications, some level of porosity is acceptable. It is common for one casting to have varying porosity zones on different part features. Each zone may have its own unique threshold based on functional needs. Sealing surfaces between machined castings or critical threaded holes might have a tighter tolerance than non-functional machined surfaces or clearance bores. Typically these zones are identified on a manufacturing print.^27^ No universal standards exist for acceptable porosity levels within castings. Threshold are set based on supplier requirements on assembled products (like sealants or o-rings), past design practice, and functional testing. These thresholds are often deemed proprietary information to original equipment manufacturers (OEMs) and are protected through non-disclosure agreements.

The binary classification process associated with specification requirements creates a problem for ML applications. Defects form along a continuous measure of size. That important detail is lost when a binary *acceptable*/*scrap* result recorded. As seen in Figure 4, a pore with a 2.1 mm size is labeled as scrap, but a pore at 1.9 mm is acceptable. The loss of fidelity on the defect measurement with a binary classification creates problems for supervised ML. Results in the data space may overlap due to this lack of distinction with a binary classification.

### Stochastic Formation of Defects

**Figure 5:**
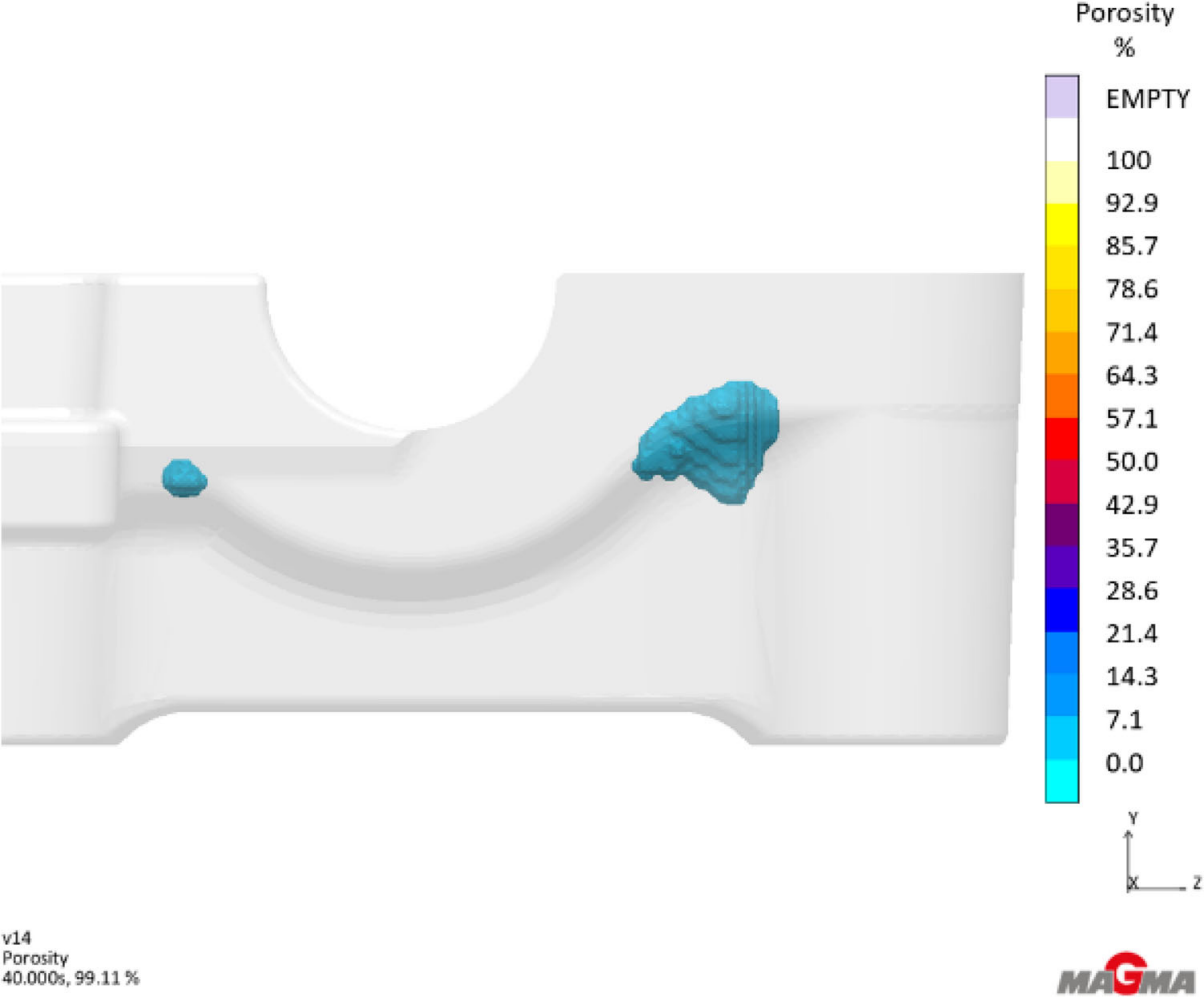
Example of simulated predicted porosity zone.

The injection and solidification of castings follows known physical rules that are modeled in casting simulation software. Rules for fluid flow, heat transfer, feeding, cooling, and many physical calculations are factored into the simulations. As a result, simulations have proven to be good at predicting locations or zones as to where porosity defects will occur during the casting process. Figure 5 shows an example of this porosity predicted zone produced by MAGMA simulation software 39.

In production, this predicted porosity zone does not create the same porosity from casting to casting. There is a stochastic, or random, nature to porosity formation within a casting. Theory says this stochastic formation occurs due to the random formation of dendrites as the metal starts to solidify which cause shrink porosity^40^,^41^ and the heterogenous nucleation sites for pores that can cause gas porosity.^24^,^40^,^41^ Oxides and inclusions are examples of these heterogenous nucleation sites for porosity that are randomly distributed through the liquid metal.^24^

**Figure 6:**
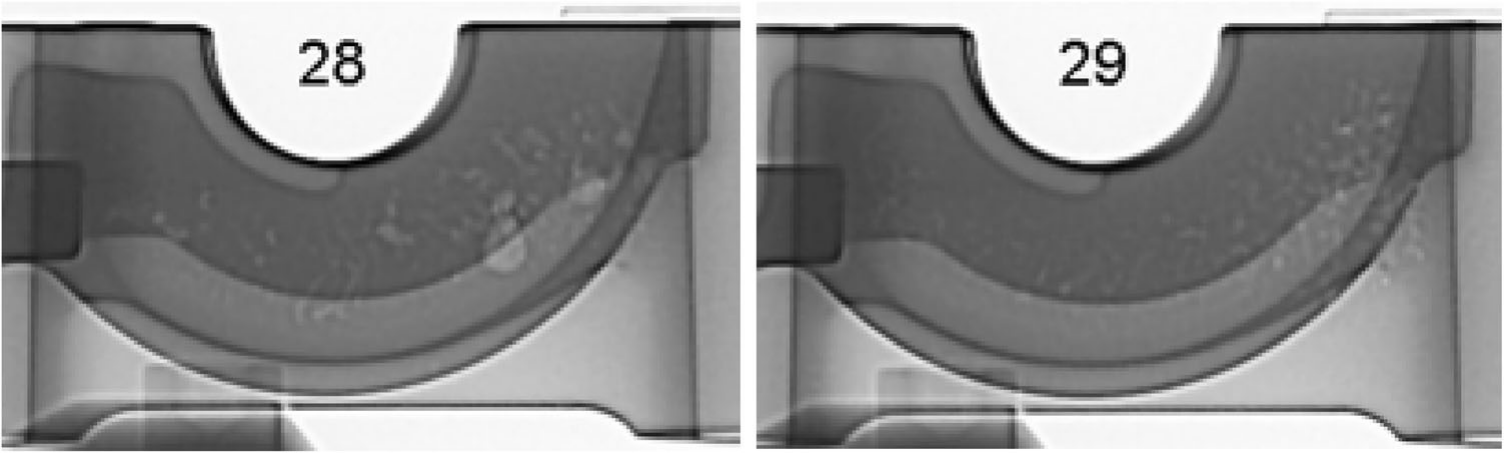
Sequential castings showing stochastic porosity formation.

This stochastic theory was shown in a recent industrial experiment.^42^ In this experiment, one hundred castings were produced with no process changes. These castings were serialized and inspected using a Bosello SRE Max with a 225 max KV power rating X-ray unit. Results showed significantly different porosity formation between castings. Figure 6 shows two castings from this experiment that were sequentially produced. The formation of the porosity was in the simulated predicted zone, as seen in Figure 5. However, the porosity was random and different between sequential castings even with no process changes. That paper showed no statistical difference in the critical process parameters between the best nine castings and the worst nine castings.

**Figure 7:**
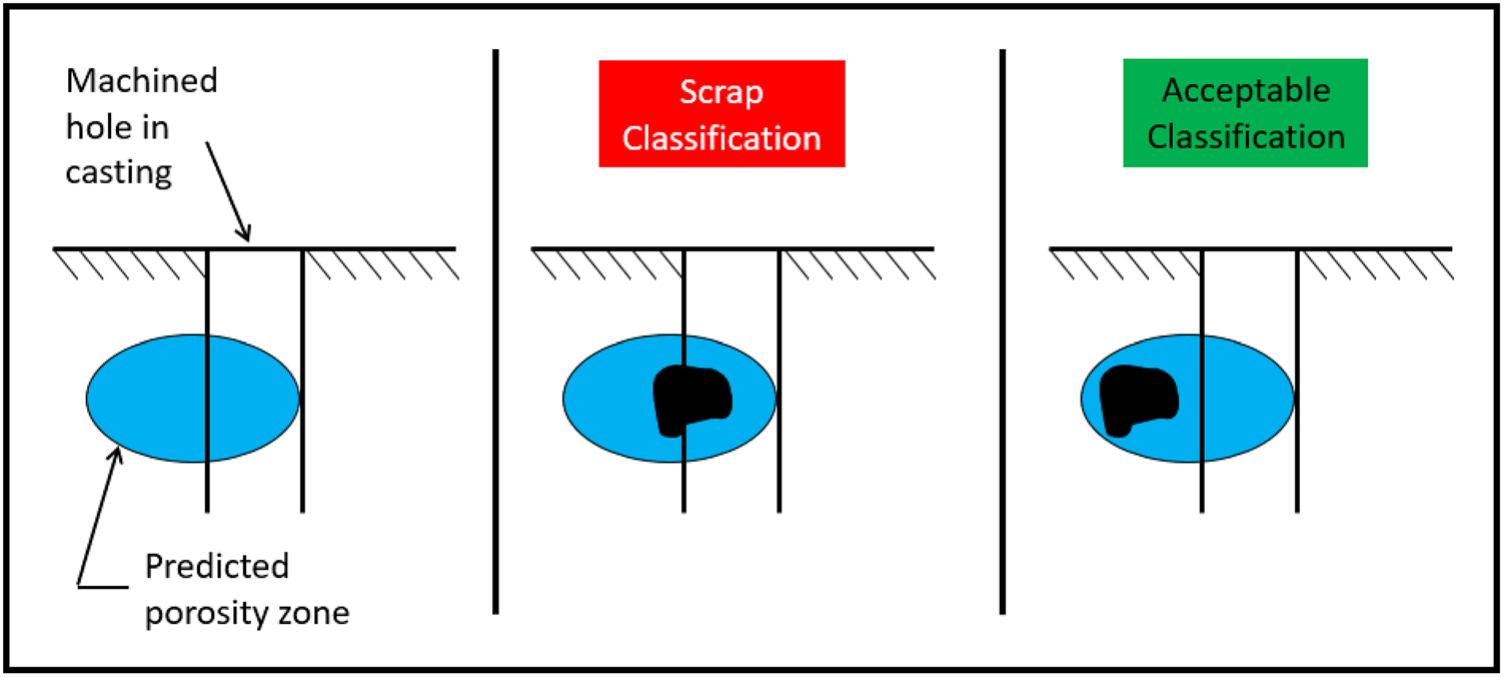
Classification issue due to stochastic defect formation example.

If the predicted porosity zone is away from any machined surface, the randomness associated with the porosity formation will have no impact on the classification of the final part. The porosity will not be uncovered or seen during visual inspection. If a hole or machined surface is cut into a zone predicted to hold porosity, the machining could potentially expose the porosity, pending random formation of the porosity. Figure 7 provides a visual example of how different stochastic porosity formations can alter classifications of the castings.

The randomness of the porosity formation directly connects to the ML problem of overlap. The data space of these points will be the same if two castings have identical input parameters. However, the random formation of the porosity causes one casting to be scrap and the other acceptable. The overlap will cause the ML algorithm to struggle and possibly fail at providing meaningful insight. The collected data amounts to noise that the ML cannot pattern.

### Secondary Process Variation

Selecting machining tolerances for a casting is a critical part of the product design. Machining tolerances are selected based on process capability, manufacturing costs, quality, life-cycle impacts, and functional requirements.^43^–^45^ There are various methods for optimizing the tolerance selection that have been studied and published.^43^–^48^

**Figure 8:**
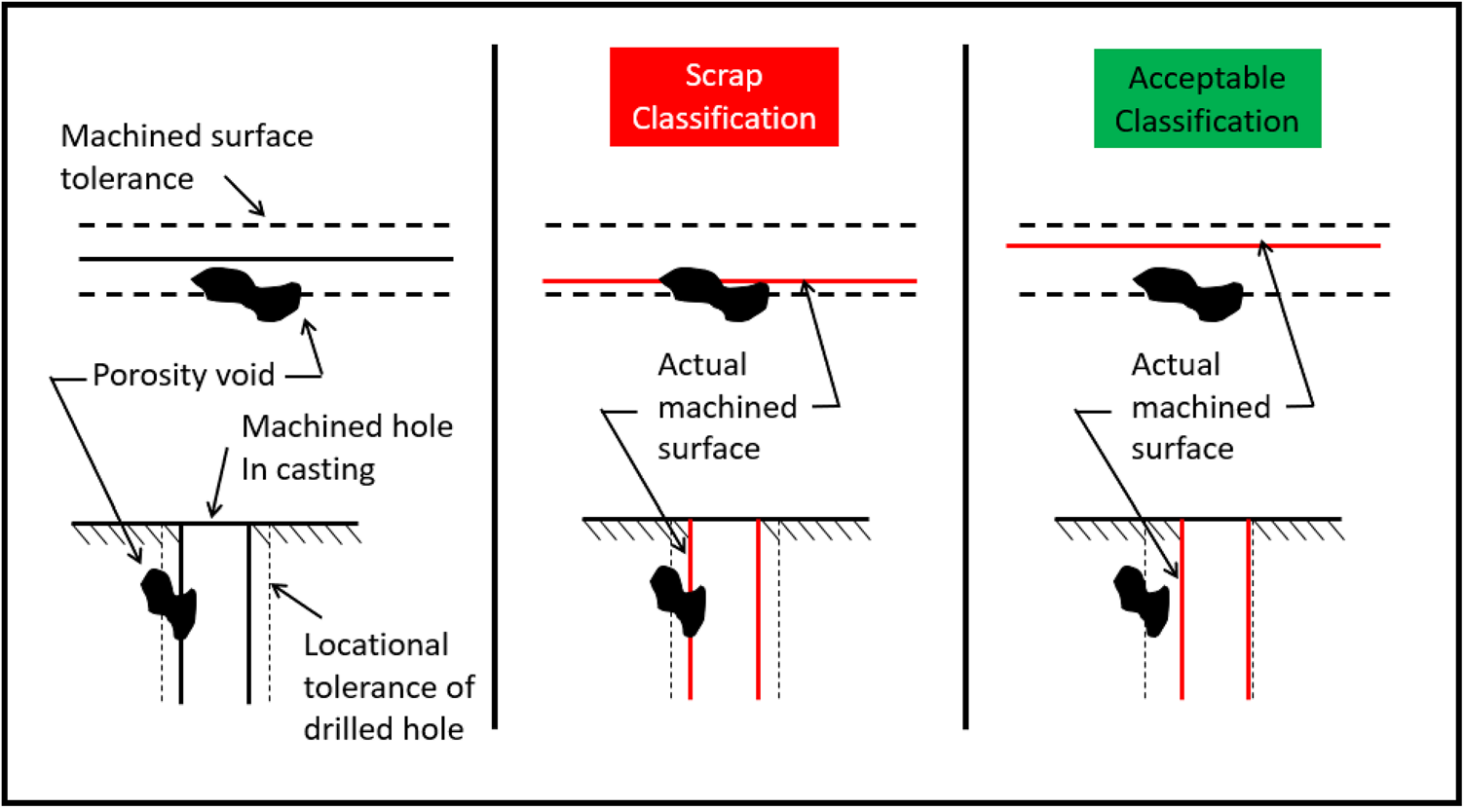
Classification issue due to secondary process variation example.

The natural variation that occurs in machining processes and the tolerances associated with the feature create allowable differences part to part. Geometric dimensioning and tolerancing (GD&T) are used on manufacturing prints to control the measurement of features. The ASME Y14.5 standard^49^ is often referenced for GD&T requirements on prints.^50^ The variation associated with the manufacturing process by the tolerancing affects the classification of casting defects. Figure 8 shows an example of the effect tolerancing and machining variation can have on defect classifications for machined surfaces and a drilled hole.

Like the random formation of defects, the variability within the processing of the part has the potential to create data space overlap. This overlap could potentially be avoided if every machined dimension was also collected and included into the algorithm. However, this additional inspection would be extremely costly to do in a production environment. Additionally, it would still not guarantee results of the ML prediction since the true condition of the part below the machined surface is still unknown. The ground truth needed to train ML algorithms for accurate predictions is not collected by traditional means within manufacturing.

### Visual Defect Inspection

The last element that affects the classification of castings is the visual inspection process. Although the technology exists for computer vision inspection in certain applications such as two-dimensional surfaces,^15^,^51^,^52^ cost and product mix within most manufacturing plants prevent this from being widely applied.^15^ Humans often complete the inspection and classification of defect results.

Much research has been published on a human’s ability to complete visual inspections on machined product. A person is capable of identifying 50–80% of defective machined products with 100% visual inspection.^14^–^19^ Considerable training on visual inspection is needed to achieve the high side of this range. This includes a comprehensive knowledge of defects, planned eye scanning paths on parts, and appropriate environmental conditions such as lighting.^15^,^53^,^54^ Many manufacturing companies do not undertake this considerable effort, even though there are large costs associated with poor quality being passed as acceptable.^14^,^15^ As a result, classification rates will be on the inferior end of the range.

The ramifications of poor classification practices because of visual inspection cannot be overlooked for supervised machine learning. Most manufacturers stay in business, because they can develop a process that is capable of a high yield. As a result, the data sets generated are highly unbalanced. Acceptable results greatly outnumber scrap results. This issue is compounded when potentially half the defective product is labeled as acceptable instead of its true scrap classification.

The adage “garbage in, garbage out” can easily be applied to supervised ML algorithms based on visual inspections. Poor visual inspection leads to incorrect classification of results. These incorrect results create data space with overlap. Overlap will cause ML algorithms to struggle to find a pattern in the noise collected within manufacturing data sets.

### Combination and Summary

The four elements described can individually contribute to misclassification of defects. Unfortunately, these elements do not act independently of each other. Instead, they combine and change through time to create more classification confusion for supervised machine learning.

**Figure 9:**
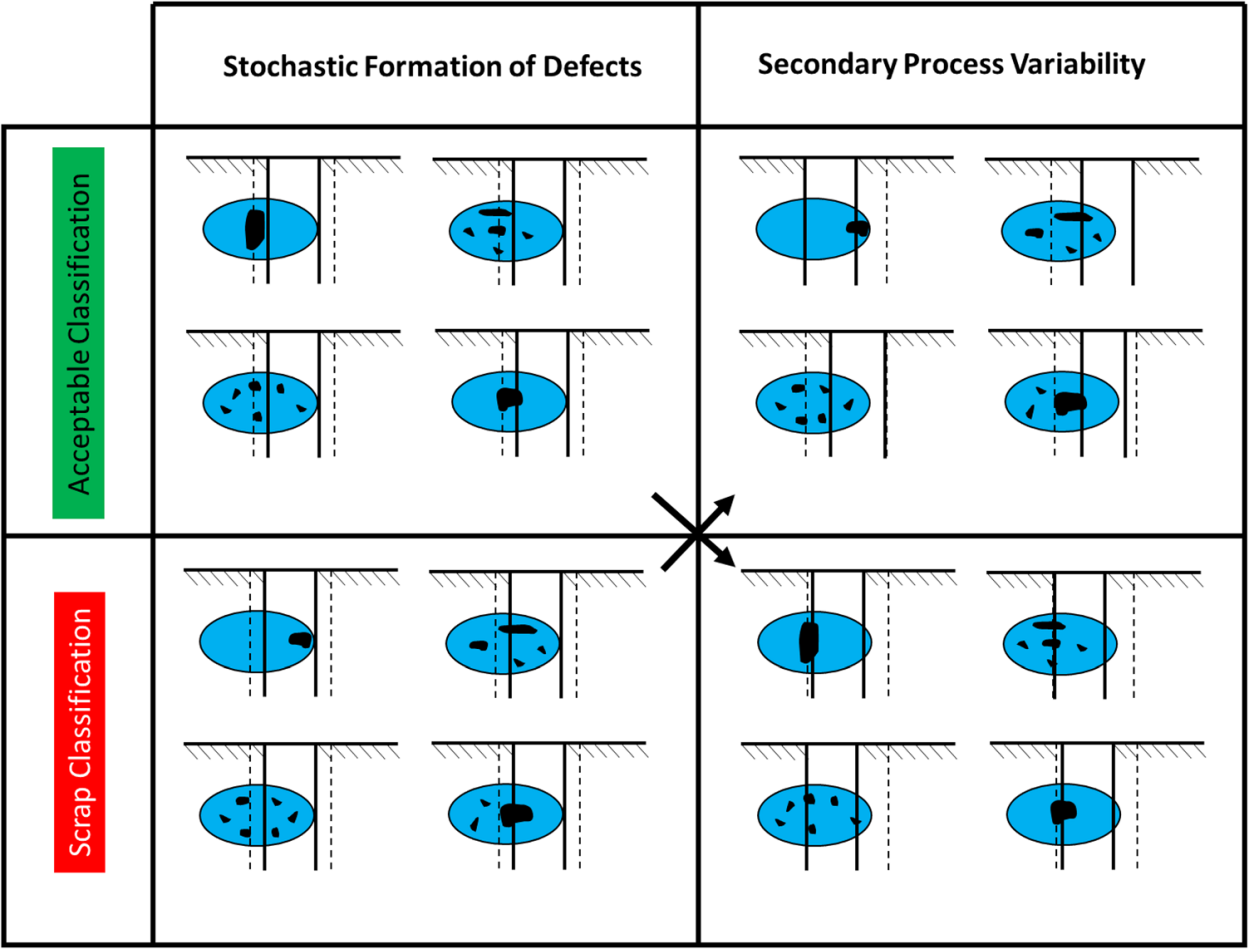
Combination of elements example.

In particular, the *Stochastic Formation of Defects* and the *Secondary Process Variation* combine to create misclassifications*.* In some combinations where the porosity forms and how it is machined, the results are classified as acceptable. In other cases, the casting may be classified as scrap. Figure 9 is created to visualize these combinations. The first column in the figure shows different levels of random porosity in the predicted porosity zone. A machined hole is drilled into the casting. In the first row, the parts are classified as acceptable based on a theoretical specification. In the second row, the parts are classified as scrap. In the second column, the random porosity formations are exchanged between the top and bottom row. Changes to the location of the hole based on machining variation are shown that make the previously acceptable parts scrap and scrap parts acceptable. The true classification is never known without a 100% X-ray inspection of each casting.

These two elements can also be combined with the *Visual Defect Inspection* to further complicate results. Visual inspection is dependent upon the person who is performing the task. Operator-to-operator performance of visual inspection can vary considerably.^14^,^15^,^54^ Proper classification becomes a probability based on the chances of porosity forming, if the machining opens it up, and whether the inspection catches the defect.

All four of these elements change through time. As previously mentioned, operators who perform the inspection task will change shift-to-shift but also will likely change with turnover. New inspectors face a learning curve of defect identification while simultaneously fighting off the repetitive nature of the work. The probability of detecting defective castings changes through time. Unfortunately, the validity of the classification results is unknown as one looks at historical data.

Specification thresholds can also change based on new suppliers or additional testing. Perhaps a different vendor can allow a slightly larger porosity specification, because its o-ring is improved. Or a new part failure has shown the product is used in ways it was not designed. Now a maximum porosity size previously accepted could be rejected. To build large data sets for ML, this data would need to be consistent through time. This knowledge is lost with the binary classification of scrap. The previous data become useless for supervised ML.

Finally, manufacturing processes vary through time. Both the casting process and machining process change based on equipment maintenance, tool wear, tool replacement, die changes, or process setting improvements. To provide a detailed example, consider tool changes in machining. Part-to-part variation in machining tolerances is often very small, given the repeatability of modern machining equipment. However, once a tool breaks and is replaced by a new tool, there is a functional change in the manufacturing system. Provided the new machined dimension falls within the designed tolerances, manufacturing will proceed without a second thought even if the new dimension is a step change from the previous tool. This changes the dynamic of the porosity exposed and the classification of the part through time.

These four elements of *Binary Acceptance Specifications, Stochastic Formation of Defects, Secondary Process Variation,* and *Visual Defect Inspection* all influence the final classification of a part. As discussed, many of these elements can create an overlap within the data space, which ML will struggle to produce meaningful predictions. This struggle can be compounded by the highly unbalanced data sets that often exist in manufacturing.

A cursory review of current operational practices would suggest manufacturers are collecting the needed data to apply ML in production manufacturing settings. However, without understanding how the ML algorithm interprets the results, the user will struggle to gain the promised value from ML technology. The industry may have very clearly defined specifications, but if it only collects acceptable/scrap and not the actual size or clustering of the void, valuable information for ML is lost. A surface is machined and then visually inspected. However, the actual truth is unknown since an operator cannot see below the surface to understand if the void still exists in the casting. Also, dimensions and locations of features are not captured on 100% of the product, so that data do not exist for ML to utilize. Manufacturers may feel good they inspect 100% of castings to ensure only good parts get to their customer but fail to realize the fallacy of human inspection rates in repetitive visual tasks.

In the end, it is not the complexity of ML technology and implementation that fails the manufacturer. Instead, the data the manufacturer have put considerable effort in to specify, create, and inspect parts to prevent the ML from being successful. The next section will provide insight into how the bias-variance tradeoff within ML is influenced by these misclassifications and the importance of a critical error threshold with highly unbalanced data that exists in manufacturing. This will help a user understand the financial impact and ML accuracy levels required for successful implementation of ML and why they often fall short in providing value in manufacturing.

## Impact on Supervised Machine Learning

Supervised machine learning algorithms are trained on input data and the known results to find patterns to accurately make predictions.^55^,^56^ There is limited ability to overcome poor training data that does not represent the prediction trying to be made. Accurate classifications are critical to successful ML applications.^57^,^58^ The impact of misclassification of results will be reviewed within this section.

### Bias-Variance Tradeoff

The Bias-Variance tradeoff graph is often used to visualize the error associated with supervised machine learning models. The generalization error of a ML model is comprised of three components: inherent error, bias, and variance, as described in Eqn. 1.^56^ The generalization error is the error produced by the model when applied to independent test data.

1 $$ {\text{Generalization\,Error}}\,{{ = }}\,{\text{Inherent\,Error}}\,{{ + }}\,{\text{Bias}}^{{{2}}} \,{{ + }}\,{\text{Variance}} $$

**Figure 10:**
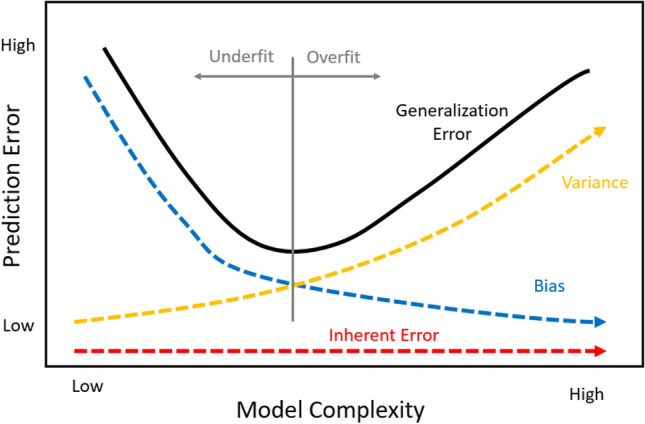
Bias-Variance tradeoff graph.

The generalization error is used to select the tuning parameters during the training of a supervised ML algorithm and even which choice of learning algorithm used for a specific data set.^56^ The goal is to avoid underfitting and overfitting to the training data. This is done by minimizing the squared bias component while ensuring the model can be generalized to future data as represented by the variance. The inherent error is error that exists based on the data used and will be discussed in detail later. This bias-variance tradeoff can be seen in Figure 10.

The accuracy and the generalization error of the ML model are related as seen in Eqn. 2 where Accuracy is the fraction of samples correctly classified, with a value of 1 representing perfect classification. When there is a high level of accuracy in the model, then there is a low generalized error rate.

2 $$ {\text{Generalized\,Error\,Rate}}\,{{ = }}\,\left( 1 -{{\text{ Accuracy}}} \right) $$

### Confusion Matrices

**Figure 11:**
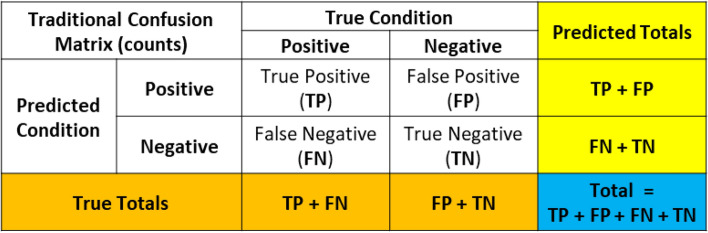
Traditional confusion matrix based on counts.

A confusion matrix is used in ML classification problems to understand how well the trained model performs. The confusion matrix shows the accuracy of the predictions made by the ML model in comparison to the true conditions of the test data. If the ML model performs well, it will correctly identify True Positives (TP) and True Negatives (TN), while only having a small number of False Positives (FP) and False Negatives (FN). The organization of the traditional confusion matrix is seen in Figure 11.

**Figure 12:**
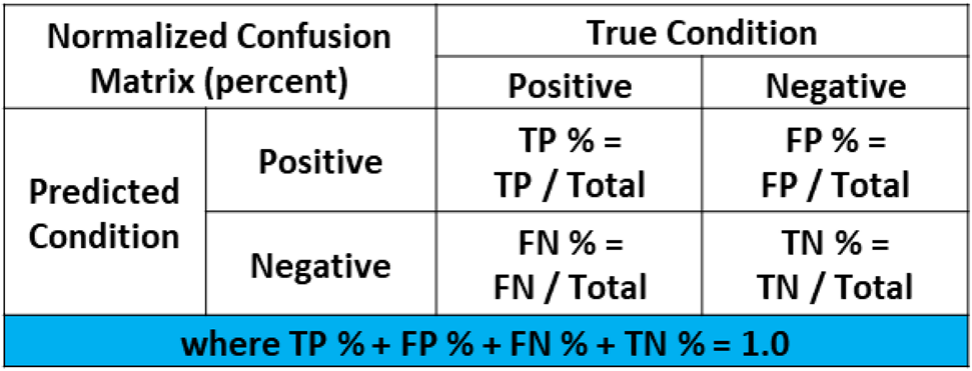
Normalized confusion matrix based on percentage.

A confusion matrix can be normalized when the individual counts, such as TP, are divided by the overall total count. This normalization process allows the user to understand the percent predictions the model makes in each of the categories. The details of the normalized confusion matrix are seen in Figure 12. This normalized percentage for the TP, FP, FN, and TN is carried forward in the balance of the equations presented in this work. The values used in equations are now labeled with a percent sign (%) to indicate they are a fraction and no longer a count. This approach was taken, because the discussion focuses on error rates and percentages and not a count as traditionally used in a confusion matrix.

**Figure 13:**
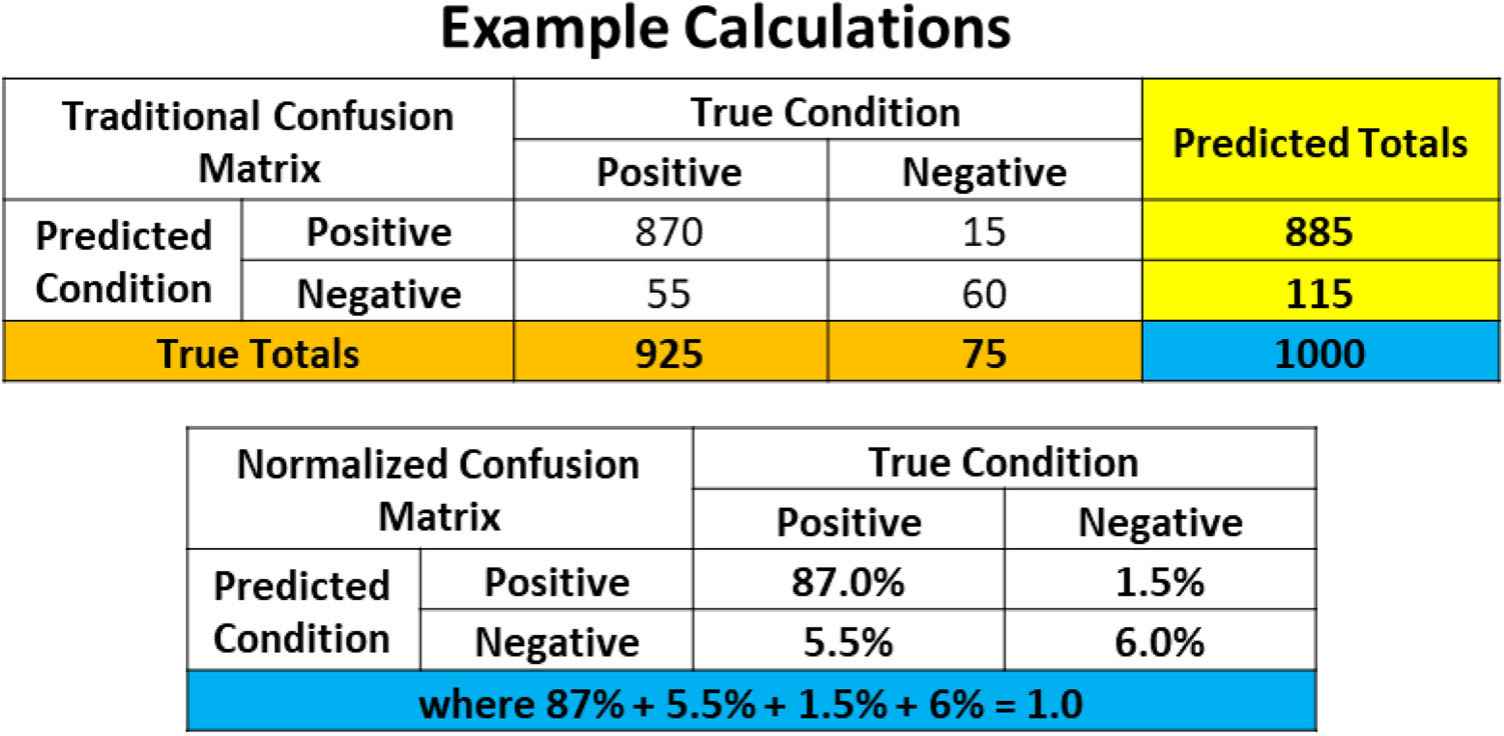
Example calculations on traditional and normalized confusion matrices.

Figure 13 is created to help illustrate a generic example of how the counts of a model as shown in Figure 11 are calculated as percentages in a normalized confusion matrix as seen in Figure 12.

**Figure 14:**
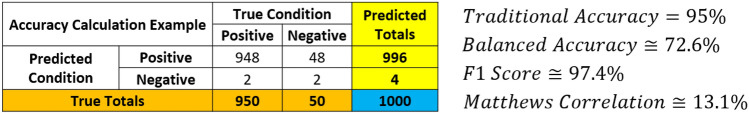
Example of different accuracy calculations.

The concept of measuring the accuracy of a ML model can be hotly debated. Multiple types of accuracy measures exist to summarize the model in different ways. Traditional accuracy, balanced accuracy, F1 score, or Matthews correlation coefficient are some of the most commonly used accuracy metrics.^59^,^60^ A shortcoming of the traditional accuracy measure is evident in highly unbalanced data. These unbalanced data sets often exist in manufacturing. A high traditional accuracy value may give a user a false sense about how well the model performs. For example, a recorded accuracy of 95% may lead one to believe the model is “good”. In fact, this could be a result of the model predicting 100% acceptable product and the product having a 5% scrap rate. In this case, the prediction provides no value, even though its 95% accurate. Other accuracy calculations provide a different perspective on the overall model accuracy for unbalanced data. A theoretical example in Figure 14 highlights the value differences in the accuracy metrics.

The best calculation of model accuracy is not for this paper to help decide. Instead, consistency must occur when comparing a decision made before and after the implementation of ML. The traditional accuracy approach is used in this work for several reasons. First, this metric was selected, because it is commonly used in industry even with its shortcoming for unbalanced data. Additionally, it is expected the ML user reviews the entire confusion matrix and not just an accuracy statistic calculated to truly understand the prediction of the ML model. Finally, the traditional accuracy creates a situation where the math is simplified and provides insight into creating the critical error threshold equation.

Prior to ML implementation, the decision automatically made in manufacturing is all product is acceptable, so there are no predicted negative conditions. The product with actual negative conditions is rejected after additional processing. These are the false positives created by assuming all product is good. After ML implementation, the costs associated with False Positives (FP%), False Negatives (FN%), and True Negatives (TN%) all must be considered. The traditional accuracy calculation for normalized confusion matrices is used and can be seen in Eqn. (3).

3 $$ \begin{aligned}   {\text{Accuracy}}\, &  = \,\frac{{{\text{TP}}_{\% }  + {\text{TN}}_{\% } }}{{{\text{TP}}_{\% }  + {\text{TN}}_{\% }  + {\text{FP}}_{\% }  + {\text{TN}}_{\% } }} \\     &  = \,\frac{{{\text{TP}}_{\% }  + {\text{TN}}_{\% } }}{1} \\ & 
= {\text{TP}}_{\% }  + {\text{TN}}_{\% } \, \\  \end{aligned} $$

where:

$$ {\text{TP}}_{\% }  = {\text{Percentage\,of\,True\,Positives\,as\,decimal}} $$

$$ {\text{FN}}_{\% }  = {\text{Percentage\,of\,False\,Negatives\,as\,decimal}} $$

$$ {\text{TN}}_{\% }  = {\text{Percentage\,of\,True\,Negatives\,as\,decimal}} $$

$$ {\text{FP}}_{\% }  = {\text{Percentage\,of\,False\,Positives\,as\,decimal}} $$

With Eqn. 1, the generalized error rate in Eqn. 2 can be simplified to focus on the incorrect predictions of the ML model as shown in Eqn. 4.

4 $$ {\text{Generalized\,Error\,Rate}} = \left( {1 - {\text{Accuracy}}} \right) = 1 - \left( {{\text{TP}}_{\% }  + {\text{TN}}_{\% } } \right) = \left( {{\text{FP}}_{\% }  + {\text{FN}}_{\% } } \right) $$

### Critical Error Threshold (CET)

**Figure 15:**
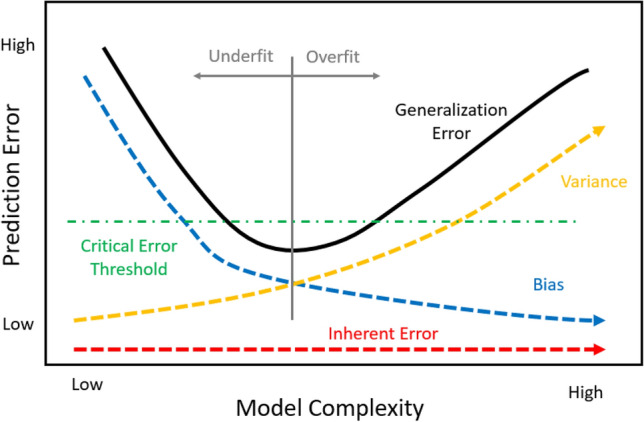
Bias-Variance tradeoff graph with critical error threshold.

A concept not found in literature to date, but of great importance to applications of ML in manufacturing, is understanding a *critical error threshold* (CET) exists for the generalized error in the bias-variance tradeoff graph. This CET defines applications of trained ML that provide value to the organization to implement. Figure 15 shows the CET updated bias-variance tradeoff graph. Financial value is achieved by the manufacturing organization if a model’s generalized error falls under the CET.

CET is a financial review of the accuracy associated with a machine learning model. The prediction accuracy of the ML model, the costs associated with the scrap, and the cost to implement must be justified when compared to current scrap costs as seen in Eqn. 5

5 $$ {\text{Total\,Scrap\,Cost\,Before\,ML}} > {\text{Total\,Scrap\,Cost\,After}}\,{\text{ML}} + {\text{Cost\,of}}\,{\text{ML}}\, $$

With details regarding specific current scrap performance, scrap costs, value-add costs, volumes, cost for ML implementation, and the percentages from the normalized confusion matrix, Eqn. 5 can be expanded to provide additional analysis in defining the CET as seen in Eqn. 6. It is important to note that the value-add costs could represent more than scrap costs added to the part before the defect is uncovered. This variable can also represent additional inspection costs, schedule adjustments, additional setups, warrantee claims, and other items that drive manufacturing costs due to defective products in the supply chain.

6 $$ \begin{aligned}    & {\text{CET}}\, \to \,{\text{Accuracy\,of\,the\,ML\,model\,in\,terms\,of\,FN}}_{\% } ,\,\,{\text{TN}}_{\% } ,\,\,{\text{FP}}_{\% } \,{\text{is\,such\,that}}: \\     & \left( {S_{\% } } \right)\left( {C_{\$ }  + V_{\$ } } \right)\left( {{\text{EAU}}} \right) > \,\left( {{\text{FN}}_{\% }  + {\text{TN}}_{\% } } \right)\left( {C_{\$ } } \right)\left( {{\text{EAU}}} \right) + \,\left( {{\text{FP}}_{\% } } \right)\left( {C_{\$ }  + V_{\$ } } \right)\left( {{\text{EAU}}} \right) + {\text{ML}}_{\$ } \,\,\, \\  \end{aligned} $$

where:

$$ {\text{CET}} = {\text{Critical\,Error\,Threshold}}\, $$

$$ S_{\% }  = {\text{Current\,Scrap}}\,\% \,\,{\text{of}}\,{\text{Casting\,as\,decimal}} $$

$$ C_{\$ }  = \,{\text{Casting\,Scrap\,Cost\,per\,part}} $$

$$ V_{\$ }  = {\text{Processing\,Value\,Add\,per\,part}} $$

$$ {\text{EAU}} = {\text{Estimated\,Annual\,Usage\,or\,Volume}} $$

$$ {\text{FN}}_{\% }  = {\text{Percentage\,of\,False\,Negatives\,as\,decimal}} $$

$$ {\text{TN}}_{\% }  = {\text{Percentage\,of\,True\,Negatives\,as\,decimal}} $$

$$ {\text{FP}}_{\% }  = {\text{Percentage\,of\,False\,Positives\,as\,decimal}} $$

$$ {\text{ML}}_{\$ }  = {\text{Annualized\,Machine\,Learning\,Implementation\,Cost}} $$

If there is no optimization within the manufacturing process with the implementation of the ML model, then the scrap rate before ML must equal the scrap rate after model implementation. As per the normalized confusion matrix, this scrap rate is the summation of the True Negative condition, regardless of prediction as shown in Eqn. 7.

7 $$ S_{\% }  = {\text{TN}}_{\% }  + {\text{FP}}_{{\% \,\,}} $$

The generalized error rate and CET relationship exist per Eqn. 8. There is motivation to implement ML in manufacturing when the generalized error rate is below the CET.

8 $$ {\text{Generalized\,Error\,Rate\,}} < {\text{CET}} $$

If no optimization has happened with the implementation of the ML model as defined in Eqn. 7, the CET value can be simplified as seen in Eqn. 9. This is accomplished by solving for the ratio between the right and left hand of Eqn. 6 and substituting the generalized error rate from Eqn. 4 and the scrap percentage as per Eqn. 7.

9 $$ \begin{aligned}    & {\text{CET}} = \,{\text{TN}}_{\% } \left( {\frac{{V_{\$ } }}{{C_{\$ } }}} \right) + FP_{\% }  - \frac{{\overline{{{\text{ML}}_{\$ } }} }}{{C_{\$ } }} \\     & {\text{assuming\,no\,scrap\,optimziation,}}\,{\text{where}}:\, \\ &    \overline{{  {\text{ML}}_{\$ } }}  = {\text{Annualized\,ML\,cost\,per\,EAU}}\,\left( {{\text{volume\,normailzed}}} \right) \\  \end{aligned} $$

The values for FN and FP will vary by tuning and training of the ML algorithm. The casting cost and value-add costs for defects are dependent on the part and processing. Slight improvements in predictions could yield financially favorable results for highly expensive value-add costs. If the value-add costs are low, the accuracy of the model is poor, or the cost to implement the ML is expensive, then value gained by implementation will not be justified. The generalized error must fall under the calculated CET for the organization to achieve a financial benefit. There is no motivation to adopt and implement ML within manufacturing if the accuracy of the model prediction does not yield financial benefits. Therefore, it is important to understand how the inherent error and squared bias affect the generalized error and implication of the CET.

### Inherent and Bias Error

**Figure 16:**
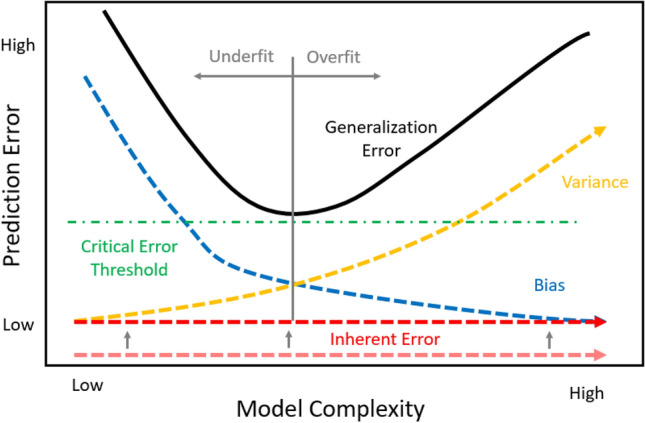
Increased inherent error.

In Eqn. 1, the inherent error exists, because the data used for the prediction do not completely represent the ability to predict the output.^56^ There likely exists additional input variable data that would improve the prediction on the model. To reduce the inherent error, these additional variables would need to be identified and included as predictors in the model. Within manufacturing, the impact of the inherent error may be large. The data collected may struggle to accurately train the model, because critical variables are not included. This would increase the inherent error, thereby increasing the generalized error of the prediction. Additionally, by not including the critical parameter, the data space that exists will likely overlap since the true cause for the results are not known. If the generalized error is increased above the CET, as seen in Figure 16, the value of the ML model is not worth the investment.

The squared bias term from Eqn. 1 is described as the amount by which the mean model estimate based on the trained data differs from the true mean.^56^ Typically, a more complex model created from the training data means a lower squared bias term. The model will be overfit to the training data. When applied to other data beyond the training set, it will perform poorly. This performance is captured in the variance term.

**Figure 17:**
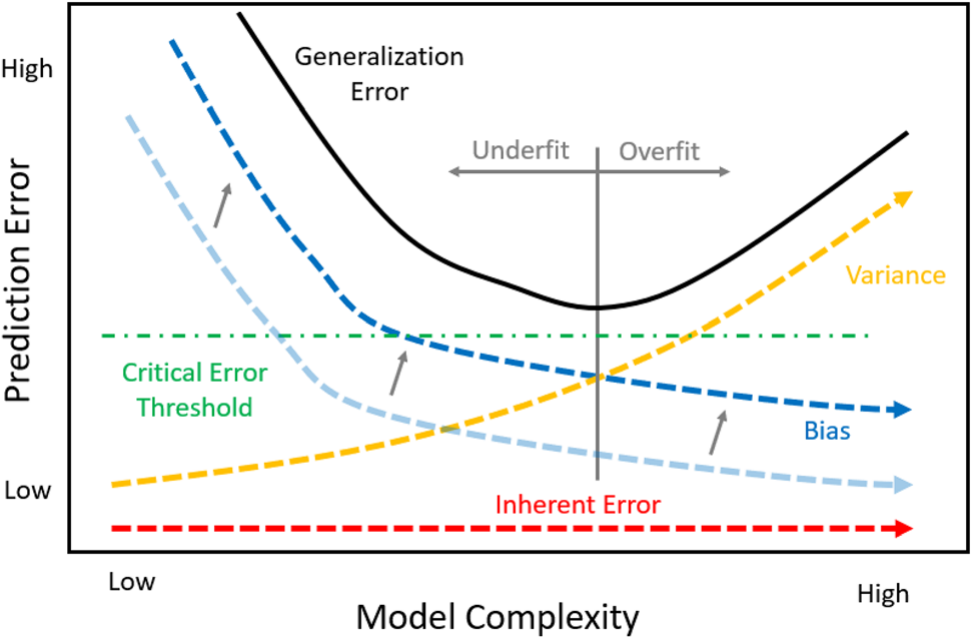
Increased bias.

The squared bias term is directly related to the four elements of classification issues identified within this paper. By misclassifying the training data, the squared bias component shifts upward, increasing the generalized error of the model. The bias term in the graph could be thought of as approaching an asymptotic limit of the misclassification rate associated with the training data. The larger the rate of misclassification within the training data, the higher the shift upward in the bias term and the resulting generalized error of the algorithm. If this shift up is substantial enough, the generalized error may exceed the CET as seen in Figure 17.

Although machine learning is a powerful tool to help find patterns, the data it is trained on must represent the critical parameters required for a good prediction to eliminate data space overlap (inherent error) and classified correctly to provide proper training (bias). The challenge for supervised ML in manufacturing is addressing both items. Data collected in manufacturing are limited and may not capture the total data space needed for an accurate prediction. The probability all the results are properly classified, especially from visual inspection, is poor. The CET calculation could provide a benefit if the ML is tuned to focus on false positives or false negatives, provided there is an economic advantage with the scrap or value-add costs.

This is further compounded by the highly unbalanced nature of manufacturing data. Consider an example in a ML algorithm created to improve the click-rate for web advertising. Success in this application could be achieved by increasing from a 2% click rate to a 5% click rate. This can also be considered as utilizing ML to move from a 98% error rate to a 95% error rate. Getting under the CET may be easy in this advertising case. Compare this to a manufacturing example where the success of product being produced is theoretically 95% with a 5% scrap rate. The goal of ML is to increase this to a near perfect prediction. Because quality yield rates within manufacturing are typically good, the ability to implement ML requires perfect data collection and process knowledge. It should not be a surprise to see this technology fail to produce meaningful results in many manufacturing settings. The generalized error rate is above the CET needed due to this imbalance of the data, missing data, and misclassified results.

## Recommendations and Future Study

The work presented here should challenge the typical approaches for classification and use of supervised ML within the foundry and manufacturing industries. A systems approach is needed to gather all data parameters within the process to minimize the generalization error. If the CET is crossed, the supervised machine learning model adds no financial value to the operation. There are three recommended areas of study or improvements needed.

### Systems Approach to Process Data (Inherent Error)

Although briefly discussed in this paper, there is a critical importance in reducing the inherent error for the model. Those interested in applying supervised ML in manufacturing must ensure the proper input variables are included in the model. The inherent error within manufacturing is likely a large portion of the generalized error. Limited data collection systems exist for the entire manufacturing system. There is a long-standing history with shot injection parameters within HPDC. However, the industry lacks commercially available or widely adopted data collection systems for thermal balance of the tooling, cycle time analysis of the entire process, and overall machine equipment performance. Even though the importance of thermal equilibrium in the tool is known,^25^ the industry is left to make assumptions and focus on what is easily measured. Quality performance of an 8% median scrap rate, as described at the beginning of this paper, highlights the results of current practices.

Correct data and process understanding is needed to reduce the inherent error. Adding variables that help pattern the prediction and eliminating variables that do not contribute will reduce the generalized error of the model. Understanding the entire die casting system is the first step to know all the process data available. A systems approach is needed to review and document all possible parameters associated with the process. There are many sub-systems within die casting creating data to be collected and studied. Some of these sub-systems include the thermals of the die, the variation in the lube spray system, and the equipment performance. This is an area of research that needs continued focus to ensure the correct variables and data are available for ML models. The data collected within the industry to date have not solved the quality problems die casting faces. Additional research is needed in this area to document all sources of variability introduced into the HPDC process.

### Four Elements of Classification Issues (Bias)

As described, there are four key elements that impact classification of casting: Binary Acceptance Specifications, Stochastic Formation of Defects, Secondary Process Variation, and Visual Defect Inspection. These misclassified results create data space overlap, thereby shifting the bias of the model and potentially making the model useless. This challenge is complicated further with the highly unbalanced data sets associated with manufacturing and the CET associated with the process.

To ensure bias is reduced, the classification of the result variables in the data collected must be accurate. This will be a challenge and must be addressed through equipment and training. An accurate model requires eliminating the binary classification of scrap. Acceptable and scrap classifications will not be sufficient. Even multiple classifications for different types and location of scrap have limited benefit given the continuous nature of the random defect formation and secondary processing.

Technologies like in-line X-ray equipment must be considered in applications where ML is applied. The bias associated with ML models would be improved by knowing the amount and location of the defect 100% of the time. This would also eliminate the delay of quality results from machining to the foundry. Additionally, it could reduce the human visual inspection of X-ray images if technologies like automatic defect recognition are used.

Without investment in X-ray equipment, the largest component of classification issues given the historical performance researched, is the visual inspection of final product. If only 50–80% of the defects are properly identified, a portion of results are being misclassified as acceptable. The ML algorithm cannot overcome this overlap. Additionally, ML is faced with challenges regarding highly unbalanced data sets from the manufacturing process. As a result, considerable effort must be given with defect inspection. Texts regarding how to improve visual inspection for castings are published and provide guidance to train and improve operators.^15^

Because humans are fallible, the inspection task should also be investigated for automation. Computer vision systems utilizing ML present opportunities to automate a large portion of this visual inspection. Even with technology limited to 2-dimensional surfaces, implementing this can reduce the required workload on human inspectors. This can allow inspectors to focus just on hard to capture areas, such as holes where visions systems may not be as successful. In addition, the resultant images gathered from these vision systems would be combined with the process data to provide additional ML opportunities.

### Unsupervised ML and Feature Importance

The challenges with supervised machine learning are considerable due to the limitations of data collection of the process (inherent error) and misclassification of training data (bias). Unsupervised ML is an approach that can provide value in manufacturing. Unsupervised ML is learned on data inputs without any knowledge of the results. It typically focuses on clustering and anomaly detection algorithms. Data processing and process control are two areas where unsupervised ML can benefit manufacturing today.

Manufacturing processes are highly complex systems. If collected, the volume and velocity of data that can be produced by equipment is extremely large. Manufacturing can produce more columns of data with the complex system than individual rows of events. In HPDC, it has been estimated that there are hundreds of thousands of possible columns due to the time-series nature of some variables used such as speed, pressure, and temperature.^61^ Often reducing these time-series down to an overall statistic loses critical information. The use of unsupervised ML to complete anomaly detection is highly advantageous. Unsupervised ML can be used to help identify when process data,^62^ images,^13^ and time-series data^63^ are outside normal ranges.

A human operator working in the manufacturing cell could not handle the level of data the cell produces. A computer with ML could manage the process control analysis and alert the operator when there are anomalies. The anomaly thresholds are set by the ML algorithm based on past performance of the process much like the control limits are set on a statistical process control (SPC) chart. Traditionally, shot monitoring system limits are human driven by past operator experience or limited developmental experimentation and can be considered a specification limit for that parameter. Unsupervised ML can take a high-dimensional SPC approach to data analytics, unlike traditional shot monitoring systems. An additional benefit of unsupervised ML anomaly detection is it can apply to time-series data sets to detect changes in the entire profile of the time-series set. Traditional shot monitoring systems only monitor statistics such as average fast shot speed calculated from those profiles. This creates the potential to miss subtle changes in the profile, such as additional braking done at the end of the shot to prevent flashing, which could have consequential implications on the casting quality.

The other machine learning application that can be utilized is feature importance. Related to supervised ML, feature importance is the process of utilizing machine learning to identify which variables have the most important influence on the prediction. Feature importance can provide an advantage even by identifying the few process parameters to investigate with a design of experiment (DOE). This DOE could optimize the process and reduce the scrap even if the ML model is incapable of creating a highly accurate model. Additional study is needed on the impact of misclassification of results on feature importance, but it appears a promising ML tool to assist manufacturing applications.

## Conclusions

Supervised machine learning is a powerful tool that has been successfully applied in many industries. There have been substantial advances in ML domains such as image classification and natural language processing. Comparing these to applications of supervised ML in manufacturing is like comparing apples and oranges. Both use ML, yet they are distinctly different and must be treated accordingly.

The challenges of supervised machine learning in manufacturing are significant due to classification issues and limitations in data collection. The four elements proposed (*Binary Acceptance Specifications, Stochastic Formation of Defects, Secondary Process Variation,* and *Visual Defect Inspection*) influence the final classification of a part. Misclassification creates data space overlap. This overlap alters the bias in the training of supervised machine learning, possibly rendering the model financially useless in a production environment. Understanding the critical error threshold provides economic guidance on when ML can be successfully applied.

Until manufacturing can establish system-wide gathering of process variables and eliminate classification issues, the success of supervised ML will be limited to highly controlled research or academic experiments. Much noise exists in the system today. This does not mean ML is to be abandoned, but instead different approaches are needed for manufacturing to see the benefit.

Beyond traditional uses of supervised ML, feature importance and unsupervised ML provide entry points for manufacturers looking to enter and start using machine learning. The potential time savings and guidance on critical input parameters feature importance can provide needs to be better understood and utilized within manufacturing. This could be a noteworthy savings in experimentation and the optimization process for die casters. Additionally, utilizing unsupervised ML for process control and anomaly detection allows for the use of machine learning in manufacturing, while creating the foundation needed for future supervised ML. This foundation is created when a company improves its classification of parts produced (reducing the bias and overlap) and optimizes the data that could improve the prediction model (reducing the inherent error). In the end, these changes will position manufacturing to benefit from accurate predictions of supervised machine learning while obtaining an improved understanding of the process.
